# Effects of Antioxidants in Reducing Accumulation of Fat in Hepatocyte

**DOI:** 10.3390/ijms19092563

**Published:** 2018-08-29

**Authors:** Jung-Pyo Yang, Ji-Hun Shin, Seung-Hwan Seo, Sang-Gyun Kim, Sang Hyung Lee, Eun-Hee Shin

**Affiliations:** 1 Department of Parasitology and Tropical Medicine, Seoul National University College of Medicine, and Institute of Endemic Diseases, Seoul 03080, Korea; fellnim@naver.com (J.-P.Y.); charisma4395@naver.com (J.-H.S.); stopsh23@naver.com (S.-H.S.); 0949kim@hanmail.net (S.-G.K.); 2 Department of Neurosurgery, Seoul National University College of Medicine, SMG-SNU Boramae Medical Center, Seoul 07061, Korea; nslee@snu.ac.kr; 3 Seoul National University Bundang Hospital, Seongnam 13620, Korea

**Keywords:** vitamin C, N-acetyl-l-cysteine, astaxanthin, oleic acid, free radical scavenging, lipogenesis

## Abstract

The progress of the hepatic steatosis (HS), a clinicopathological status, is influenced by cellular oxidative stress, lipogenesis, fatty acid (FA) oxidation, and inflammatory responses. Because antioxidants are gaining attention as potent preventive agents for HS, we aimed to investigate anti-lipogenic effects of the antioxidants vitamin C (VC), N-acetylcysteine (NAC), and astaxanthin (ATX) using hepatocytes. For this, we established an in vitro model using 1 mM oleic acid (OA) and human liver hepatocellular carcinoma (HepG2) cells; 10 μM antioxidants were evaluated for their ability to reduce fat accumulation in hepatocytes. Our results showed that all three antioxidants were effective to reduce fat accumulation for the molecular targets such as reduction in lipid droplets, triglyceride (TG) concentration, reactive oxygen species (ROS) production, and cell apoptosis, as well as in gene expressions of endoplasmic reticulum (ER) stress-related effectors, lipogenesis, and inflammatory cytokines. There were simultaneous increases in diphenyl-1-picrylhydrazyl (DPPH) radical scavenging effect, cell survival, AMPK phosphorylation, NRF2-related gene expression for cellular defense, and FA β-oxidation. However, among these, ATX more effectively inhibited ER stress and lipogenesis at the intracellular level than VC or NAC. Consequently, ATX was also more effective in inhibiting cell death, lipotoxicity, and inflammation. Our result emphasizes that ATX achieved greater lipotoxicity reduction than VC and NAC.

## 1. Introduction

Metabolic syndrome (MS) is defined by the presence of metabolic alterations that increase an individual’s risk of developing type 2 diabetes mellitus (T2DM) and cardiovascular disease [1,2]. Patients with MS commonly have nonalcoholic fatty liver disease (NAFLD) that develops due to the presence of three major risk factors: obesity, T2DM, and hepatic lipid accumulation [1,2,3,4]. Furthermore, aging is a risk factor for NAFLD in premenopausal women [5]. In men, increasing age is associated with increased visceral fat accumulation [6]. The liver plays a central role in whole body lipid homeostasis including glycolysis and lipogenesis [7]. Lipogenesis (called de novo lipid biosynthesis) occurs when excess carbohydrates are consumed or when the circulating insulin levels are high [7]. In contrast, in cases of low insulin levels and high glucagon levels, FA oxidation or lipolysis occurs for the mobilization of FA and liver uptake [7]. Insulin resistance is associated with lower availability of lipoprotein lipase and contributes to hypertriglyceridemia [1,8]. Cholesterol and FA are accumulated in the liver of patients with NAFLD through this mechanism; this is accompanied by oxidative stress and reactive oxygen species (ROS) formation [9]. These ROS induce lipid peroxidation and trigger tumor necrosis factor TNF-α-regulated liver damage [8,9]. Until recently, popular approaches involved the use of therapeutic components for complications, such as hepatic fat accumulation, insulin resistance, inflammation, and fibrosis, or the administration of micronutrients with antioxidative and anti-inflammatory effects to prevent and treat NAFLD [9]. For example, vitamins (C, D, and E), resveratrol, and astaxanthin (ATX) have been used as micronutrients and antioxidants for NAFLD management [2,8,9,10,11,12]. Similarly, N-acetylcysteine (NAC), a small protein with clinically effective health benefits, is also used as an antioxidant and detoxifier for the clinical intervention of NAFLD [10,13,14]. Vitamin D and E are fat soluble vitamins, inhibit lipid oxidation, and associated with the lipid and glucose metabolism in the liver suggesting the potential therapy for NAFLD [3,8]. Regarding the functional mechanism, NAC plays a role in blocking the propagation of lipid peroxidation in the liver and improving liver function [13]. Vitamin C (VC), a water-soluble vitamin, acts as a chain-breaking antioxidant capable of scavenging essentially all the physiologically relevant free radicals [2]. ATX, a natural antioxidant carotenoid occurring in several living organisms, has a positive effect on cardiovascular disease, obesity, and dyslipidemia as a quencher of ROS/RNS single- and 2-electron oxidants and a chain-breaking scavenger of free radicals [12]. Each of these natural and synthetic compounds, VC, NAC, and ATX, has been tested for protective effects on HS and NAFLD; however, they have never been simultaneously compared.

Increasing attention has been focused on protective and supplementary therapies for these metabolic disorders because of the associated major risk of mortality in the elderly and the financial burden for their prevention and control. Thus, we conducted a preliminary cell study to select the antioxidants with superior efficacy for HS alleviation before performing an in vivo study, using three kinds of antioxidants with clinical potential: VC, NAC, and ATX, with clinical potential. In detail, the molecular mechanisms that indicate HS alleviation are presented by changes in the radical scavenging effect, cellular lipid contents, triglyceride (TG) concentration, endoplasmic reticulum (ER) stress effectors, NRF2-related antioxidation effectors, ROS level, apoptosis, expression of genes involved in lipogenesis and FA β-oxidation, AMPK phosphorylation, NF-κB signaling, and inflammatory signals. We believe that our study is significant in identifying antioxidants with the potential of alleviating HS before using further clinical measures and is meaningful in that it simultaneously compares various molecular targets for confirming the general anti-HS effects.

## 2. Results

### 2.1. Induction of Cellular Steatosis in HepG2 Cells

**Figure 1:**
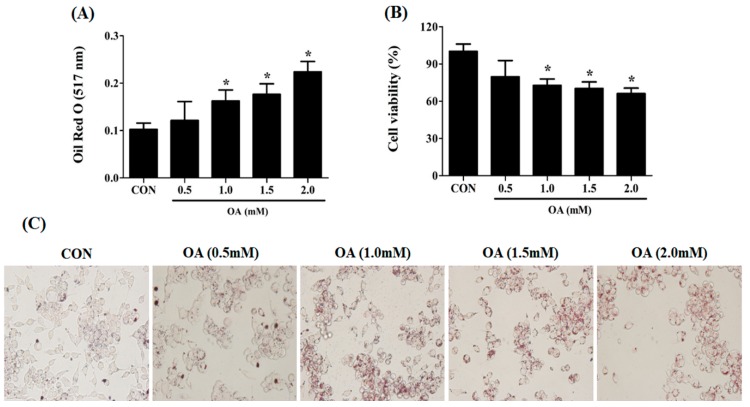
Induction of cellular steatosis in HepG2 cells. An appropriate concentration of OA for inducing cellular steatosis was determined using ORO staining and cell counting assay (CCK-8). Lipid content of the cells was measured at 517 nm after ORO staining (**A**). The viability of the OA-treated cells was determined at various concentrations of OA solution using the CCK-8 kit (**B**). The morphological distribution of lipid droplets within the cells was observed using ORO staining (**C**). Data are represented as mean ± standard deviation (SD) (*n* = 4) values. Asterisk (*) indicates a significant difference compared with the control (*p* < 0.05).

To establish an in vitro model of hepatic cellular steatosis, HepG2 cells were treated with OA solutions of various concentrations (0.5, 1.0, 1.5, and 2 mM). The lipid contents of the cells were examined by absorbance at 517 nm after ORO staining (Figure 1A). The control was serum-free DMEM media containing 0.1% DMSO and 1.0% BSA, similar to the culture media of the experimental groups. Cellular steatosis was successfully induced with a statistical difference in the absorbance compared with that in the control when the OA solution was treated by a concentration >1.0 mM (0.1 ± 0.001 vs. 0.16 ± 0.02 at 517 nm) (Figure 1A). In contrast, the cell viability was significantly decreased with treatment of OA solution >1.0 mM concentration compared with the control (72.7 ± 5.1 and 100 ± 6.0, respectively) (Figure 1B). The result presented in Figure 1A also confirms the microscope observations that lipid droplets formed within the cells are shown in red colors (Figure 1C). Accordingly, we determined that the most appropriate OA concentration was 1 mM because the treatment of 1 mM OA solution was relatively less toxic despite effective steatosis induction in the cells.

### 2.2. Reduction in the Intracellular TG Levels and Lipogenesis in OA-Treated Cells after Antioxidant Treatment

**Figure 2:**
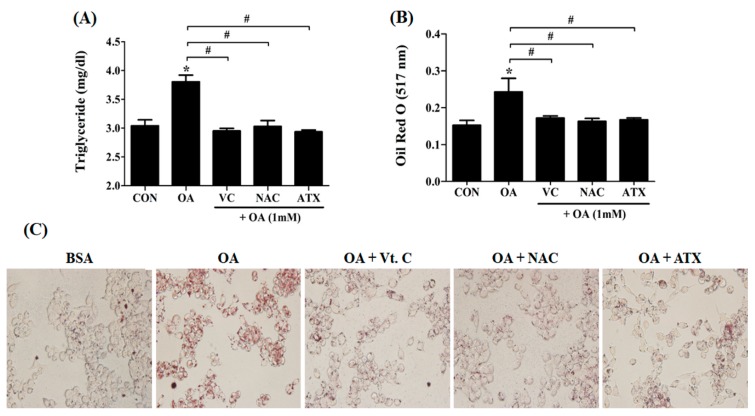
Intracellular triglyceride (TG) levels and lipid synthesis after antioxidant treatment in the OA-treated cells. When 1 mM OA solution and 10 μM antioxidants were added, the TG concentration (**A**) and lipid contents (**B**) within the cells were examined using the TG assay kit and ORO staining method. At this time, lipid droplets stained with ORO solution appear in red color (**C**). Data are represented as mean ± SD values (*n* = 4). Asterisk (*) indicates a significant difference compared to control (*p* < 0.05). Sharp (#) indicates a significant difference among the experimental groups (*p* < 0.05).

The following study focused on whether the antioxidant treatment can reduce lipogenesis. TG, a major component of the lipid droplets, is an important marker used for evaluating the degree of FA formation. In our results, the TG level (mg/dL) in the OA-treated cells was significantly reduced following antioxidant treatments (3.8 ± 0.1 vs. 2.9 ± 0.03 to 3.0 ± 0.1) (Figure 2A). Similarly, ORO staining results showed decreased lipogenesis (Figure 2B) and decreased lipid droplets after antioxidant treatments (Figure 2C). It is noteworthy that the effect of antioxidants on the reduction of lipogenesis was similar, irrespective of the type of antioxidant used.

### 2.3. Free Radical Scavenging Effect of Antioxidants on DPPH Radical as Well as ROS Resulted from Cellular Steatosis

**Figure 3:**
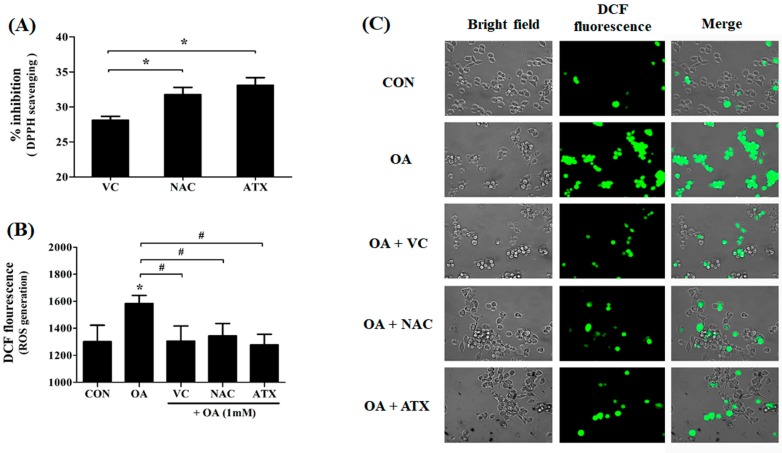
Effects of antioxidants in scavenging the DPPH radicals and ROS generation in the OA-treated cells. DPPH radical scavenging effect (%) of each antioxidant was compared by percentages based on the control level (**A**). The ROS level produced after treatment with antioxidant in OA-treated cells was determined using DCF-DA staining (**B**). DCF-DA-stained cell images (**C**). Data are represented as mean ± SD values (*n* = 4). Asterisk (*) indicates a significant difference compared with the control (*p* < 0.05). Sharp (#) indicates a significant difference among the experimental groups (*p* < 0.05).

To investigate the free radical scavenging effect of antioxidants resulting from cellular steatosis, we examined the DPPH radical scavenging effect (%) and ROS generation (DCF fluorescence) following antioxidant treatment in OA-treated cells (Figure 3A,B). The DPPH assay shows the DPPH radical scavenging activity of the antioxidant itself (Figure 3A), and DCF-DA staining shows the fluorescence for ROS (Figure 3B). Our results show that the antioxidant treatment in OA-treated cells scavenged the DPPH radicals as follows: ATX (33.1% ± 0.9%) > NAC (31.8% ± 1.2%) > VC (28.1% ± 0.3%), suggesting the difference in the free radical scavenging activity of the antioxidant itself (Figure 3A). The DPPH radical scavenging effect of ATX was significantly higher and the greatest among all three antioxidants. Similarly, the scavenging effect of antioxidants for ROS was determined using DCF-DA staining (Figure 3B). The antioxidant treatment inhibits ROS generation in OA-treated cells; however, the suppression ability of the three antioxidants to suppress ROS generation was not considerably different (Figure 3B). Thus, our results show that VC, NAC, and ATX are commonly used effective agents for scavenging of free radicals. In addition, ATX treatment was most efficient.

### 2.4. Effects of Antioxidants to Gene Regulation of ER Stress Due to Steatosis

**Figure 4:**
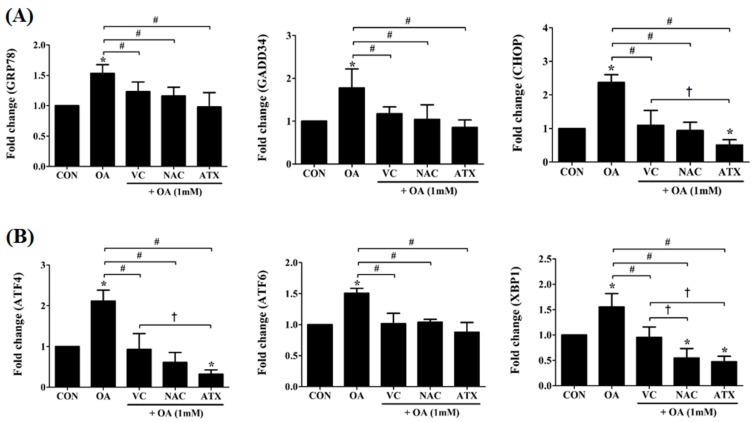
Changes in the ER stress-related genes after antioxidant treatments in the OA-treated cells. The expression levels of *GRP78*, *GADD34*, *CHOP*, *ATF4*, *ATF6*, and *XBP1*, major effectors in the cellular process of cell death due to ER stress were measured using real-time PCR. Data are represented as fold changes compared to the mRNA level in control and are shown as mean ± SD values (*n* = 6). Asterisk (*) indicates a significant difference compared with the control (*p* < 0.05). Sharp (#) indicates a significant difference among the experimental groups (*p* < 0.05). Dagger (†) indicates a significant difference compared to each other among the antioxidants, VC, NAC, and ATX, using one-way ANOVA (*p* < 0.05).

To understand the effect of antioxidants in overcoming oxidative stress owing to cellular steatosis, we observed the changes in several genes indexing the ER stress in the cells (Figure 4). ER stress, a condition characterized by the accumulation of misfolded and unfolded proteins results from ROS-derived oxidative stress and induces cell apoptosis. For ER stress-derived cytosolic and nuclear effectors, we selected several genes, such as glucose-regulated protein 78 (*GRP78*), activating transcription factor 6 (*ATF6*), activating transcription factor 4 (*ATF4*), X-box binding protein 1 (*XBP1*), growth arrest and DNA damage-inducible protein (*GADD34*), and C/EBP homologous protein (*CHOP*) (Figure 4). The proteins encoded by these genes are involved in the unfolded protein response, and their synthesis is markedly induced under conditions leading to the accumulation of unfolded polypeptides in ER. Among them, the ER chaperone, *GRP78*, and the cytosolic components, *CHOP* and *GADD34*, are important for the ER stress-mediated apoptosis pathway (Figure 4A), and their transcription factors, *ATF4*, *ATF6*, and *XBP1*, mediate the cellular ER stress through the transcription of ER molecules (Figure 4B). As shown in Figure 4, the gene expressions of ER stress-effector molecules (*GRP78*, *CHOP*, and *GADD34*) in OA-treated cells were increased to the range of 1.5 ± 0.1- and 2.4 ± 0.2-fold changes; further, the gene expressions of their transcription factors (*ATF4*, *ATF6*, and *XBP1*) were also increased to 1.5 ± 0.1- to 2.1 ± 0.3-fold changes. However, the antioxidant treatment reduced the expression levels of these genes to lower than the control level (1-fold). In particular, VC treatment reduced the expression levels of six genes to the range of 0.9 ± 0.4- and 1.2 ± 0.2-fold changes, NAC to the range of 0.5 ± 0.2- and 1.2 ± 0.1-fold changes, and ATX to the range of 0.3 ± 0.1- and 1.0 ± 0.2-fold changes. The increased gene expression indicates an environment of persistent ER stress; therefore, the action of the antioxidants in the present study suggests that the reduction in the ER stress was attributable to cellular steatosis. The reducing effect of ER stress was increased by the following order: VC < NAC < ATX.

### 2.5. Nrf2-Related Gene Expression for Cellular Defense Mechanisms against ROS

**Figure 5:**
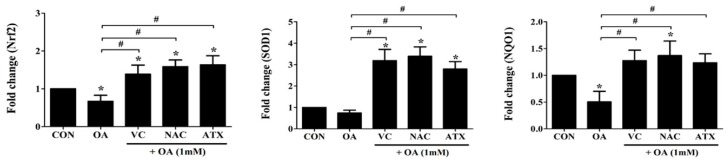
Nrf2-related gene expressions for cellular defense mechanisms against ROS. Nrf2, a transcription factor as a master regulator of the cellular redox homeostasis, and *SOD1* and *NQO1*, *Nrf2* downstream target genes, were investigated using real-time PCR analysis. Data are represented as fold changes compared to the mRNA level in control, and shown as mean ± SD values (*n* = 6). Asterisk (*) indicates a significant difference compared with the control (*p* < 0.05). Sharp (#) indicates a significant difference among the experimental groups (*p* < 0.05).

Nuclear factor erythroid 2-related factor 2 (*Nrf2*), super oxide dismutase-1 (*SOD*1), and quinine oxidoreductase-1 (*NQO*1) genes regulate the cellular resistance to oxidants (ROS) by regulating the cellular redox balance. This study examined the expressions of these genes using real-time PCR following treatment with the antioxidants, VC, NAC, and ATX, in OA-treated cells (Figure 5). The genes were decreased to the range of 0.5 ± 0.2- and 0.7 ± 0.1-fold in OA-treated cells and conversely increased to the range of 1.2 ± 0.2- and 3.4 ± 0.4-fold after the antioxidant treatment compared with that after control (1-fold). Thus, our results show that antioxidant treatment increases the expressions of Nrf2 as well as NRF2-related genes (*SOD1* and *NQO1*) that play a role in the cellular defense mechanisms against ROS.

### 2.6. Effects of Antioxidants on Cell Apoptosis Resulting from OA-Induced Steatosis

**Figure 6:**
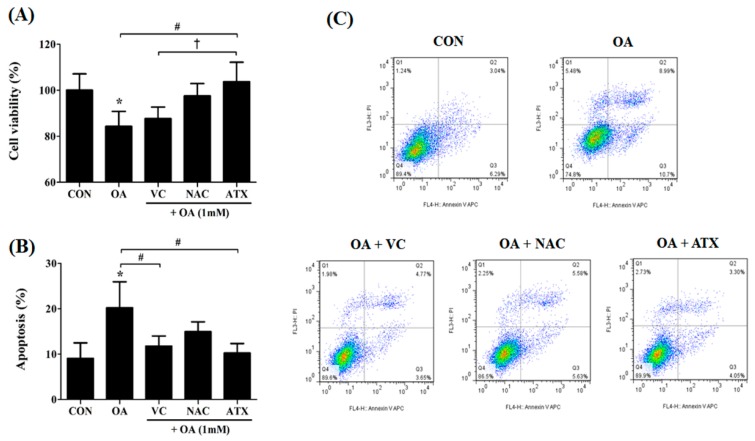
Effects of the antioxidants on cell apoptosis resulting from OA-induced steatosis. The cell viability (%) after antioxidant treatments in the OA-treated cells was investigated using the CCK-8 kit (**A**), and apoptotic cells (%) were counted by the sum of the percentages of annexin V-stained cells and dual stained cells of Annexin V and PI using FACS analysis (**B**). Gating images for the apoptotic cells are shown in (**C**). Data are represented as mean ± SD values (*n* = 4). Asterisk (*) indicates a significant difference compared with the control (*p* < 0.05). Sharp (#) indicates a significant difference among the experimental groups (*p* < 0.05). Dagger (†) indicates a significant difference compared with the each other among the antioxidants, VC, NAC, and ATX, using one-way ANOVA (*p* < 0.05).

Our results show that antioxidant treatment can reduce the oxidative stress via cellular steatosis by regulating the genes involved in ER stress and cellular defense regulation against ROS. Regarding this, we determined whether antioxidant treatment can practically reduce cell apoptosis resulting from OA treatment (Figure 6). Compared with the control, the OA-treated cells decreased the ratio of viable cells to 84.3% ± 6.5%, whereas antioxidant treatment increased it to the range of 87.6% ± 5.0% and 103.7% ± 8.5% (Figure 6A). Among the antioxidants, ATX completely recovered the decrease in the viable cells after OA treatment to the control level and blocked the effect of steatosis on the cells (Figure 6A). In contrast, the ratio of apoptotic cells (%) was significantly increased following OA treatment (18.0 ± 4.5%) compared with control (8.1 ± 3.4%), and the ratio further significantly decreased after antioxidant treatment (10.6 ± 2.4% to 14.9 ± 2.7%) compared with OA-treated cells (Figure 6B). This effect was greater in ATX than in VC and NAC (Figure 6A,B). These results are well presented in the scattered fluorescence images (Figure 6C). Thus, our results demonstrated that VC, NAC, and ATX exert direct effects in lowering cell apoptosis resulting from OA-induced steatosis.

### 2.7. Effect of Antioxidants on AMPK Phosphorylation Mediating the Cellular Adaptation to Stress Factors Such as Free Radical Accumulation

**Figure 7:**
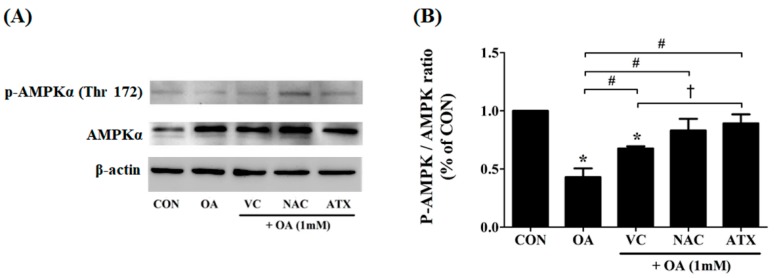
Effect of the antioxidants on AMPK phosphorylation after OA treatment. Cellular steatosis decreases AMPK phosphorylation (**A**), and the result is represented by the ratio of p-AMPK/AMPK (**B**). Data are represented as mean ± SD values (*n* = 3). Asterisk (*) indicates a significant difference compared with the control (*p* < 0.05). Sharp (#) indicates a significant difference among the experimental groups (*p* < 0.05). Dagger (†) indicates a significant difference compared with each other among the antioxidants, VC, NAC, and ATX, suing one-way ANOVA (*p* < 0.05).

Steatosis is a major causal factor inducing cellular stress conditions, such as ROS production and ER stress-related apoptosis. As a resistive mechanism, AMPK is activated to enable a concomitant inhibition of FA synthesis and activation of adenosine triphosphate (ATP)-producing catabolic pathways, including FA oxidation. In particular, AMPK phosphorylation in the liver is important because the liver is an important organ that regulates whole body glucose homeostasis. Moreover, AMPK contributes to the suppression of glycolytic and lipogenic gene expression in the liver. In this regard, we speculated the effects of antioxidants on OA-treated cells as follows. Antioxidants may increase AMPK phosphorylation to inhibit lipid synthesis in OA-treated cells. To demonstrate this, we investigated the degree of AMPK phosphorylation (p-AMPK) using immunoblotting (Figure 7A); the expression ratio of p-AMPK/AMPK (%) was calculated using ImageJ program (*n* = 3) (Figure 7B). Our results showed that OA treatment on the cells decreased the ratio of p-AMPK/AMPK (0.43 ± 0.06%) compared with controls; however, antioxidant treatment significantly increased the degree of AMPK phosphorylation (0.68 ± 0.01% to 0.89 ± 0.06% for the ratio of p-AMPK/AMPK). In particular, ATX exerted the highest effect on AMPK phosphorylation among all three antioxidants. Thus, the present study shows the effectiveness of antioxidants in increasing AMPK phosphorylation in OA-treated cells.

### 2.8. Effect of Antioxidants on Transcription Factor and Metabolic Enzymes Related to Hepatic Lipogenesis in OA-Treated Cells

**Figure 8:**
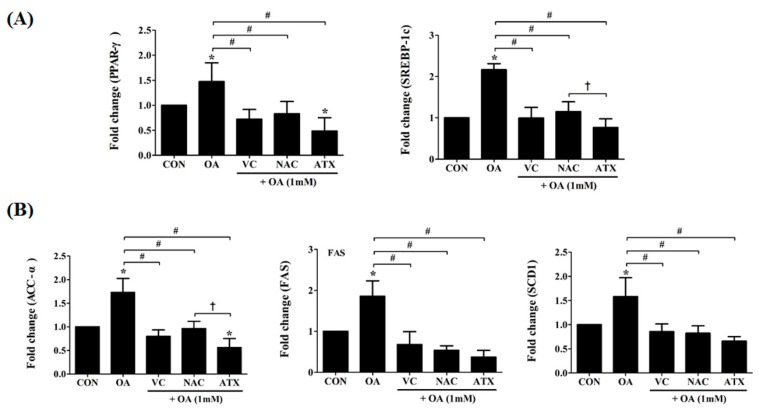
Effects of the antioxidants on the gene expressions of transcription factors and metabolic enzymes related to hepatic lipogenesis. As key transcription factors that regulate hepatic lipogenesis and FA oxidation, gene expressions in *PPAR*-γ and *SREBP-1* were investigated using real-time PCR (**A**). *ACC-α*, *FAS*, and *SCD1*, as critical enzymes promoting the biochemical conversion of glucose into FA and TG, were also investigated by gene expressions (**B**). Data are represented as mean ± SD values (*n* = 6). Asterisk (*) indicates a significant difference compared with the control (*p* < 0.05). Sharp (#) indicates a significant difference among the experimental groups (*p* < 0.05). Dagger (†) indicates a significant difference compared with the each other among the antioxidants, VC, NAC, and ATX, using one-way ANOVA (*p* < 0.05).

In the presence of intracellular lipid biosynthesis, there is a significant increase in lipid peroxides, promoting apoptosis and cellular toxicity. To evaluate the impact of antioxidants on this, we examined the gene regulation of two transcription factors that regulate hepatic lipogenesis and FA oxidation, peroxisome-proliferator-activated receptors (*PPAR-γ*) and sterol-regulatory-element-binding protein-1c (*SREBP-1c*) (Figure 8A). Moreover, for critical enzymes related to the biochemical conversion of glucose into FAs and TG, *ACC-α*, *FAS*, and *SCD-1* were examined (Figure 8B). Our result showed that antioxidant treatment with VC, NAC, or ATX reduced the expressions of these transcription factors. Thus, the gene expressions of *PPAR-γ* and *SREBP-1c* in OA-treated cells were increased 1.5 ± 0.4- to 2.2 ± 0.1-fold compared with the control, whereas they were decreased 0.5 ± 0.3- to 1.2 ± 0.2-fold compared with control after antioxidant treatment (Figure 8A). In addition, the enzymes, acetyl CoA carboxylase (*ACC-α*), FA synthase (*FAS*), and stearoyl CoA desaturase-1 (*SCD-1*), for fatty acid biosynthesis related to hepatic lipogenesis were also investigated using real-time PCR (Figure 8B). In our study, these enzymes were increased after OA treatment (1.6 ± 0.4- to 1.9 ± 0.4-fold change), whereas they decreased after the antioxidant treatment (0.4 ± 0.2- to 1.0 ± 0.2-fold change) (Figure 8B). Among the three antioxidants, ATX exerted the greatest anti-lipogenic effect.

### 2.9. Changes in Several Genes to Increase FA Oxidation after Antioxidant Treatment

**Figure 9:**
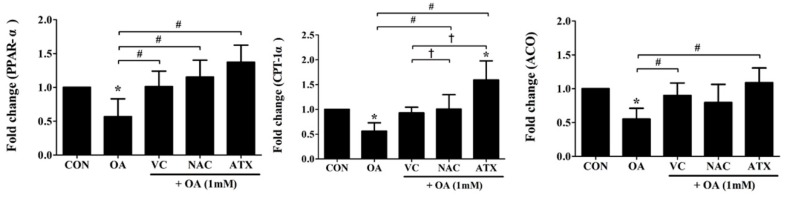
Effects of antioxidant treatments on several genes related to FA oxidation in the OA-treated cells. *ACO* and *CPT-1α*, as β-oxidation-inducing enzymes, and their transcription factor, *PPAR-α*, were examined using real-time PCR for gene expressions. Data are represented as mean ± SD values (*n* = 6). Asterisk (*) indicates a significant difference compared with the control (*p* < 0.05). Sharp (#) indicates a significant difference among the experimental groups (*p* < 0.05). Dagger (†) indicates a significant difference compared with each other among the antioxidants, VC, NAC, and ATX, using one-way ANOVA (*p* < 0.05).

Regarding the decrease in lipogenesis transcription factors by antioxidants as shown in Figure 8, β-oxidation-inducing enzymes, such as acyl-CoA oxidase (*ACO*) and carnitine palmitoyl transferse 1 (*CPT1*), and their transcription factor, peroxisome proliferator-activated receptor α (*PPAR-α*), were also investigated (Figure 9). *PPAR-α* increases FA catabolism toward peroxisomal and mitochondrial FA β-oxidation; therefore, increases in these genes after antioxidant treatment indicate that antioxidants can directly regulate both the decrease of lipogenesis and the increase of FA β-oxidation. In detail, the gene expressions of *PPAR-α*, *ACO*, and *CPT1* were decreased after OA treatment (0.55 ± 0.16- to 0.57 ± 0.26-fold change) compared with the control, whereas ATX treatment increased the expressions of these genes (1.09 ± 0.22- to 1.59 ± 0.38-fold change) to a greater extent than VC and NAC (0.80 ± 0.27- to 1.15 ± 0.25-fold change) (Figure 9). In particular, the increase of *CPT-1α* gene was the greatest among antioxidants with a statistically significant difference (1.6 ± 0.4-fold change). This result emphasizes the importance of antioxidants in reducing FA β-oxidation.

### 2.10. Reduction in the Gene Expressions of Inflammatory Cytokines as Well as NF-κB Signal after Antioxidant Treatments in OA-Treated Cells

**Figure 10:**
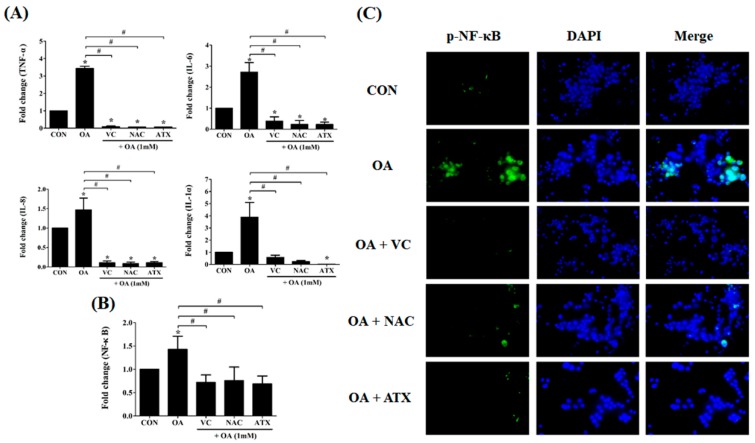
Reduction in the gene expressions of the inflammatory cytokines and *NF-κB* signal after antioxidant treatments in the OA-treated cells. *IL-1α*, *IL-6*, *IL-8*, and *TNF-α*, as cytokines inducing inflammatory responses, were examined using real-time PCR for their gene expressions (**A**). The expression of *NF-κB*, as a transcription factor inducing inflammatory cytokines, was also investigated using real-time PCR (**B**) and NF-κB-immunostaining (**C**). Data are represented as mean ± SD values (*n* = 6). Asterisk (*) indicates a significant difference compared with the control (*p* < 0.05). Sharp (#) indicates a significant difference among the experimental groups (*p* < 0.05).

According to the above results, antioxidant treatments are expected to relieve cellular steatosis by reducing lipid synthesis, ROS production, and ER stress. In addition, another factor indicating the reduction of cellular steatosis is the decrease in the inflammatory response and *NF-κB* signal. In this respect, we observed the changes in the gene expressions of inflammatory cytokines in OA-treated cells. As shown in Figure 10A, *IL-1α*, *IL-6*, *IL-8*, and *TNF-α* were commonly increased in OA-treated cells (1.5 ± 0.3- to 3.4 ± 0.1-fold change) compared with that in the control; in contrast, they decreased after antioxidant treatment (0.1 ± 0.01- to 0.4 ± 0.2-fold change), suggesting that antioxidants play a role inhibiting the inflammatory responses in OA-treated cells. Further, all the three antioxidants demonstrated a comparable effect in inhibiting inflammatory responses. The induction of anti-inflammatory response can be confirmed by the reduction in *NF-κB* signaling (Figure 10B). In this regard, we demonstrated that OA treatment induced the increase of *NF-κB* gene expression (1.4 ± 0.3-fold change) compared with the control, whereas all antioxidant treatments reduced *NF-κB* gene expressions (0.7 ± 0.2- to 0.8 ± 0.3- fold change) (Figure 10B). This result can also be demonstrated using visual immunofluorescence detection (Figure 10C). The green color in OA-treated cells indicates *NF-κB*-expression within the cells, whereas the fluorescence with green color was not shown in the antioxidant-treated cells, suggesting the down-regulation of *NF-κB* signals. These results mean that the antioxidant treatment to OA-treated cells inhibits apoptosis and inflammatory response via a cascade reaction through a decrease in the ER stress by free radical scavenging effects and the inhibition of lipogenesis by increased FA β-oxidation.

## 3. Discussion

The liver plays an important role in regulating the whole body metabolism of energy nutrients [15]. When accompanied by obesity, NAFL, also known as hepatic steatosis (HS), is characterized by excess TG accumulation in the hepatocytes [1,15]. A normal liver maintains the balance between lipid input and output by regulating the delivery of dietary fat into the liver, de novo lipogenesis, uptake of free FAs from the adipose tissue, and the formation and secretion of very low-density lipoprotein [1,15,16]. However, dysregulation of these homeostatic pathways in the liver leads to excess TG accumulation in the hepatocytes, known as liver steatosis (NAFL) [15]. HS is characterized by fat accumulation in the hepatocytes; abnormal fat accumulation causes lipotoxicity called cellular toxicity [17]. The abnormality of fat metabolism and induction of lipotoxicity lead to the development of NASH; T2DM and obesity are major risk factors for advanced NASH [18].

Recent attention has focused on the control of pathogenesis for fatty liver diseases using micronutrient antioxidants and/or dietary components, such as vitamins, ATX, NAC, resveratrol, carotenoid, curcumin, betaine, and polyphenols [2,8,9,10,11,12,13,15]. Among them, polyphenols are a heterogeneous class of plant derived compounds, with some proven hepatoprotective effects [19]. Vitamins C and E are known to react with ROS to block the propagation of radical reactions in oxidative stress-related situations [2,4]. NAC, a precursor of glutathione, acts as an antifibrotic and antioxidant agent by supplying cysteine for the hepatic glutathione synthesis that decreases oxidative stress [4,14,20]. In addition, ATX is a natural antioxidant carotenoid present in several living organisms, and simultaneously currently much interest in biological active compounds [15,21,22]. ATX is known as a nutritional supplement with antioxidant effects in various aspects, such as anticancer, antihypertensive, neuroprotective, and anti-liver fibrosis effects [15,21,22]. The common characteristics of these antioxidants include resisting oxidative stress and having the following metabolic effects as potential therapeutic agents of fatty liver diseases: decreases lipid peroxidation, HS, and hepatic lipogenic gene expressions [4,10,11,14,15,22,23]. However, thus far, the effects of antioxidants in alleviating fatty liver diseases have not been compared among several antioxidants or factors related to NAFL management simultaneously [4,10,11,14,15,22,23]. For comparing the effect of antioxidants in managing NAFL, in vivo experiments are required; however, this involves the limitation to do determining anti-steatosis effect for cells due to a lot of complementary interventions of in vivo. In this respect, our study aimed to preferentially define finely and at various angles the difference in the effect reducing fat accumulation in hepatocytes, depending on the type of antioxidant (VC, NAC, and ATX). In particular, these three antioxidants differ in their mode of action; the results may be very interesting when they are compared simultaneously. 

We chose VC, NAC, and ATX as antioxidants in our study because they are dietary supplements, are well-known, easy to use by people, and have different modes of action in natural and compound forms. In this regard, VC, a vitamin found in food and an essential nutrient, is a chain-breaking antioxidant capable of essentially scavenging all the physiologically relevant free radicals; simultaneously, it serves as a donor of reducing equivalents in multiple enzymatic reactions [2]. NAC is an antioxidant and is a cysteine-donor that acts directly and/or by increasing intracellular GSH, the most abundant cellular thiol antioxidant, particularly in the hepatic tissue; this thiol redox state is crucial for optimizing the protective ability of the cell to counter oxidative stress [14]. ATX belongs to a wide range of biologically active compounds derived from natural sources, particularly *Haematococcus pluvialis*, and is used as a nutritional supplement for antioxidant and anticancer agents, for its protective effects against diabetes and cardiovascular diseases, and its action in neuroprotection and immunity enhancement [22,24]. To simultaneously compare the antioxidant effects on these three kinds of antioxidants, we prepared an in vitro HS model using HepG2 cells and OA solution due to the increase of OA in the hepatic free FAs in patients with NAFL [17]. To investigate the anti-steatosis effect, we have performed research from various perspectives. In addition, VC and NAC were often compared with each other as antioxidant supplements for NAFLD; however, to our knowledge, ATX, a potential protector of liver function, has not been compared with the other two antioxidants for its effect in managing NAFL [23,25,26].

As expected, OA treatment to HepG2 cells successfully induced HS; at this time, all three antioxidants reduced DPPH radicals and ROS production. The increase in ROS induces ER oxidative stress; therefore, the decrease in ROS production seems to obviously accompany the reduction of the following ER stress markers, GRP78, GADD34, CHOP, ATF4, AFT6, and XBP1, which are molecular targets of ER stress investigated in our study [23,27,28]. In our results, the increases in the ER stress markers and their transcription factor caused cell death [28]; subsequently, increases in the antioxidant markers, such as NRF2, SOD1, and NQO1 are accompanied by antioxidant treatment [29,30]. In particular, NRF2 is an emerging regulator of cellular resistance to reactive oxidants and as a critical transcription factor for regulating the expression of various cytoprotective genes, such as *SOD* and *NQO1*, in several type of cells and tissues [29,30]. Accordingly, our result definitely shows that the antioxidants, VC, NAC, and ATX, provide cellular resistance to ROS in OA-treated cells through the induction of NRF2 signaling and inhibition of the ER stress marker genes. As another mechanism related with liver steatosis, the hepatic metabolism of energy nutrients, such as FA oxidation and lipogenesis is important. In this regard, medical interest in the AMPK system has recently increased with evidences that AMPK mediates some effects of the fat cell-derived adiponectin and antidiabetic drugs, prevents obesity-induced NAFLD, and inhibits SREBP-1c-mediated HS [31,32,33]. Our results also showed that the phosphorylation of AMPK (p-AMPK) was increased, PPAR-γ related with lipid accumulation was decreased, and PPAR-α related with FA β-oxidation was increased. Thus, we can conclude that VC, NAC, and ATX play equal roles in controlling lipid metabolism. Among them, ATX has a relatively more effective anti-steatosis effect than the other two antioxidants with respect to the inhibition of lipid production and the induction of FA β–oxidation, as implied in previous results that ATX is a PPAR-α agonist and PPAR-γ antagonist [24]. There is a characteristic cytokine pattern in liver steatosis such as the increase in TNF-α and IL-6 [34]. As per the mechanism, ROS and products of lipid peroxidation increase TNF-α and cause cell death, inflammation, and fibrosis [34]. In addition, IL-1, IL-6, and IL-8 are also increased in common liver diseases, such as simple steatosis, NASH, and liver cirrhosis [32,35]. If antioxidants play a role in relieving HS, inflammatory cytokines have to be reduced eventually [32,34,35]. In this regard, our results confirmed the reduction in the levels of the inflammatory cytokines, TNF-α, IL-1α, IL-6, and IL-8 after antioxidant treatment. The reduction effect in the gene expressions of inflammatory cytokines was similar for all the antioxidants. Therefore, we believe that all the three antioxidants, consequently and to the same extent, reduced the lipid contents in the OA-treated cells, although there was a difference in the functional target of detailed anti-steatosis.

Based on our results, the anti-steatosis effect of VC, NAC, and ATX was comparable in terms of the decreases in the lipid droplets and TG concentration in the HepG2 cells. However, ATX exerts a better effect on several functional targets of detailed anti-steatosis, such as decrease in ROS generation, cell apoptosis, ER stress and lipogenesis marker genes, lipid metabolism-related genes, inflammatory cytokine and NF-κB genes, and NF-κB subunit p65-immunostained expression; further, it increases the free radical scavenging effect, antioxidant genes, AMPK phosphorylation, and FA β-oxidation. ATX more effectively inhibits ER stress as well as lipogenesis at the intracellular level than VC and NAC. Furthermore, ATX consequently inhibits cell death and inflammation more effectively, suggesting reduced lipotoxicity. Regarding the mechanism, we believe that the difference between the results for functional targets of detailed anti-steatosis was caused by the differences in the functional action of the antioxidants, although precise mechanisms cannot be discussed here. Thus, the main functional mechanisms of VC, NAC, and ATX differ as follows: a donor of reducing equivalents in multiple enzymatic reactions, cellular thiol antioxidant, and biologically active compound with antioxidant function, respectively. Using the advantage of ATX, which has a broad functional action as a biological active compound, in near future, it is necessary to demonstrate the superiority of ATX, i.e., the inhibition of fat reduction as well as lipotoxicity, as a dietary supplement for anti-steatosis through an in vivo study. However, it is noteworthy that VC, NAC, and ATX are all effective antioxidants for intracellular simple fat reduction.

Our result emphasizes that the reduction in lipotoxicity is greater by ATX than by VC and NAC, although all three antioxidants play a similar role in simple fat reduction.

## 4. Materials and Methods

### 4.1. Cell Culture 

HepG2 cells, a human liver cancer cell line, were purchased from the Korean Cell Line Bank (KCLB, Seoul, Korea) and cultured with Dulbecco’s Modified Eagle’s Medium (DMEM) (WELGENE Inc., Gyeongsan-si, Korea) containing 10% fetal bovine serum (FBS, WELGENE Inc.) and 1% antibiotic antimycotic solution (WELGENE Inc.) in 100 mm dishes (SPL Life Sciences, Pocheon-si, Korea) at 37 °C in a 5% CO_2_ incubator. Cells were sub-cultured every 2 days for in vitro experiments and moved to a 96-well plate of 2 × 10^4^ cells/well, and further experiments were conducted. 

### 4.2. Preparation of Antioxidants

VC, NAC, and the esterified form of ATX were purchased from Sigma-Aldrich (St. Louis, MO, USA). VC, NAC, and ATX were dissolved in dimethyl sulfoxide solution (DMSO, Sigma-Aldrich) to a concentration of 10 mM. Thereafter, 10 mM antioxidant DMSO solution was dissolved in serum-free DMEM media to a final concentration of 1 mM. To determine an appropriate concentration of VC, NAC, and ATX for further experiments, antioxidants were prepared at three concentrations, 1 mM, 100 μM, and 10 μM, and investigated for their free radical scavenging activity and cell survival using diphenyl-1-picrylhydrazyl (DPPH) assay (Sigma-Aldrich) and Cell Counting kit-8 assay kit (CCK-8, Dojindo, Japan). The final concentration of the antioxidants in our study was 10 μM for all three antioxidants based on the criteria for high free radical scavenging activity and low toxicity for cells (Supplementary Data).

### 4.3. Induction of Steatosis Using Oleic Acid in HepG2 Cells

Oleic acid (OA) was purchased from Sigma-Aldrich and used to induce steatosis in HepG2 cells, as describe previously [36]. OA solution was prepared by adding bovine serum albumin (BSA, Fraction V, fatty acid-free, Sigma-Aldrich) in a ratio of 1:4, respectively. Briefly, when HepG2 cells were approximately 80% confluent after being cultured in 10% FBS-supplemented DMEM for 24 h in a 96-well plate (SPL Life Sciences), the supernatant was discarded, and the cells were serum-starved for 24 h before steatosis induction. Thereafter, the cells were treated with 200 μL OA solution (0–2.0 mM) for 24 h to investigate the toxicity of OA on the cells.

### 4.4. Cell Viability of Steatosis-Induced HepG2 Cells 

To observe the cell viability after treatment with OA and/or antioxidant, cells seeded as 2 × 10^4^ cells/well in a 96-well culture plate were cultured in a serum-free medium for 24-h starvation and treated with 1 mM OA and/or 10 μM antioxidant. Then, the supernatant was discarded, and cell survival was examined using CCK-8. Absorbance was read at 490 nm using a plate reader (Infinite^®^ 200 PRO, TECAN, Mannedorf, Switzerland), and cell viability was calculated as follows:

(1) $$(A_{Sample\;}-A_{blank})/\left(A_{Control}-A_{blank}\right)\;\times 100\%$$

(Control, serum-free DMEM with cells; Blank, serum-free DMEM without cells).

### 4.5. Oil-Red O Staining to Confirm Steatosis within the Cells (Histological Study)

The Oil-Red O (ORO, Sigma-Aldrich) staining method was used to confirm steatosis induction in the cells. After the supernatant of the cells treated with OA solution for 24 h was discarded, cells were fixed with 100 μL fixative solution (paraformaldehyde, Yakuri pure chemicals Co., Uji, Japan) for 10 min and stained with ORO solution that was diluted with DW to 60% for 30 min. After being washed five times in phosphate-buffered saline (PBS, Sigma-Aldrich), the cells were observed under a microscope (Olympus, Shinjuku, Japan) (×40). At this time, the control was cultured in an OA-free medium containing BSA. Experiments with antioxidant treatment were performed by a co-culture of 1 mM of OA solution and 10 μM of each antioxidant for 24 h. To quantify the intensity of ORO within the cells, the absorbance at 517 nm was monitored using a spectrophotometer (Infinite^®^ 200 PRO, TECAN, Mannedorf, Switzerland).

### 4.6. Measurement of Triglyceride Concentration in Steatosis-Induced Cells

The TG concentration was quantified using a commercial colorimetric assay kit (Cayman Chemical, Ann Arbor, MI, USA) according to the manufacturer’s instructions. Steatosis-induced and antioxidant-treated HepG2 cells were collected and centrifuged at 1500× *g* for 10 min at 4 °C. Then, the cell pellet was suspended with 1 mL cold diluent provided in the kit, sonicated for 10 s, and finally diluted 10 times with the diluent in the kit. The 10-μL samples were mixed with 150 μL enzyme solution in the kit, and incubated at room temperature (RT) for 15 min. The absorbance was measured at 530 nm using a microplate spectrophotometer (Infinite^®^ 200 PRO).

### 4.7. DPPH Radical Scavenging Activity

The free radical scavenging activities of VC, NAC, and ATX were measured using DPPH assay, as described previously [37]. Briefly, 100 μL of 0.1 mM 2-DPPH radical solution (DPPH, Sigma-Aldrich) diluted in methanol (JT Baker Chemical Co., Phillipsburg, NJ, USA) and 10 μL of 10 μM antioxidant dissolved in DMSO were mixed and incubated for 30 min in the dark. The free radical scavenging activity of each antioxidant was measured at 517 nm using a microplate spectrophotometer. Reduction in the absorbance indicated reduction in the amount of free radicals in the sample. The DPPH radical scavenging activity of each antioxidant was calculated using the following formula:

(%) inhibition ratio = [(Abs_control_ − Abssample)/Abs_control_] × 100. (2)

### 4.8. Detection of ROS in Steatosis-Induced HepG2 Cells Using DCF-DA

HepG2 cell were seeded by 2 × 10^4^ cells/well in a 96-well clear bottomed black plate (BD Falcon, Bedford, MA, USA); then, 1 mM of OA solution and 10 μM of each antioxidant were treated for 24 h. ROS produced in the cultured cells were stained with 2′,7′-dichlorofluorescin diacetate (DCF-DA), a cell-permeable probe (Sigma-Aldrich). The cells were stained with 100 μL DCF-DA diluted in PBS (20 µM) at 37 °C for 1 h in the dark. After washing with PBS, the fluorescence was measured using a microplate spectrophotometer (Infinite^®^ 200 PRO) at an excitation of 495 nm and emission of 535 nm; thereafter, observations were made using fluorescent microscopy (Leica DMI6000B microscope, ×40, TECAN, Mannedorf, Switzerland).

### 4.9. Real-Time Polymerase Chain Reaction (PCR) Analysis of Gene Expressions Regulated in Antioxidant-Treated and Steatosis-Induced HepG2 Cells

Total ribonucleic acid (RNA) was extracted from HepG2 cells using HiGene™ Total RNA prep kit (BIOFACT, Daejeon, Korea), and it was reverse-transcribed to cDNA using a Reverse-Transcription Master Premix kit (ELPIS Biotech). Thereafter, cDNA samples were subjected to real-time PCR analyses using SYBR Green PCR Master Mix (TOPreal™ qPCR 2× PreMix, SYBR Green with high ROX, BIOFACT, Daejeon, Korea), as per the manufacturer’s instructions. The expression of glyceraldehyde 3-phosphate dehydrogenase (GAPDH) gene in each sample was evaluated as an internal control. Table 1 shows the primer sequences for real-time PCR analysis of various genes (*GRP78*, *ATF6*, *ATF4*, *XBP1*, *CHOP*, *GADD34*, *Nrf2*, *SOD1*, *NQO1*, *PPAR-γ*, *SREBP-1c*, *ACC-α*, *FAS*, *SCD-1*, *PPAR-α*, *ACO*, *CPT-1*, *NF-κB*, *TNF-a*, *IL-1*, *IL-6*, and *IL-8*).

### 4.10. Flow Cytometry Analysis to Determine ER Stress-Related Apoptosis in Steatosis-Induced HepG2 Cells 

Flow cytometry was used for the quantitation of apoptotic cells by the co-staining of annexin V and propidium iodide (PI). Briefly, after 2 × 10^6^ cells/well of a 96-well culture plate were treated with OA (1 mM) and/or antioxidant (10 μM) for 24 h, the cells were harvested for APC-labeled annexin V and PI staining and subjected to fluorescence-activated cell sorting (FACS) analysis. The harvested cells were incubated with 100 μL FACS staining buffer (1% BSA and 0.1% sodium azide) containing 5 μL APC-labeled annexin V (BioLegend, San Diego, CA, USA) and 2 μL PI (Sigma, St. Louis, MO, USA) for 15 min at RT in the dark. The stained samples were analyzed using a FACSCalibur flow cytometer (Becton Dickinson, San Jose, CA, USA). Data acquisition and analyses were performed using “Cell-Quest pro” (Becton Dickinson), and further data analyses for forward/side scatter gates were performed using FlowJo software (Tree Star, Inc., Ashland, OR, USA). Data of apoptosis (%) were displayed as the percentage of cell numbers stained with annexin V alone (Q3-plot) and dual staining annexin V + PI (Q2-plot) (*n* = 4).

### 4.11. Western Blotting

Total proteins were extracted from the HepG2 cells treated with OA and/or antioxidant using the M-PER Mammalian Protein Extraction Reagent (Thermofisher, Waltham, MA, USA) and quantified with a Nanodrop spectrophotometer (Nanodrop Technologies, Oxfordshire, UK). Proteins were separated using sodium dodecyl sulfate-10% polyacrylamide gel electrophoresis at 100 V for 110 min; thereafter, they were transferred to a nitrocellulose membrane (Bio-Rad Laboratories, Hercules, CA, USA) using the Mini Trans-Blot^®^ Electrophoretic Transfer Cell (Bio-Rad Laboratories) at 80 V for 100 min. The membranes were then incubated with TBS-T with 5% skim milk (Becton Dickinson, Sparks, MD, USA), and then incubated with primary antibody which is either one of rabbit Phospho-AMPK alpha-1-, AMPK alpha-1-polyclonal antibody (Invitrogen, Waltham, MA, USA), or mouse anti-beta-Actin (Santa Cruz Biotechnology, Santa Cruz, CA, USA). As secondary antibodies, chicken anti-rabbit IgG-HRP (Santa Cruz Biotechnology) and goat anti-mouse IgG-HRP (Santa Cruz Biotechnology) were used. Signals were detected by exposing the membrane to enhanced chemiluminescence HRP substrate (Thermofisher Scientific) using a Fuji LAS1000 Lumino Image Analyzer (Fujifilm Corporation, Tokyo, Japan) and calculated using an image calculator, ImageJ program.

### 4.12. Immunofluorescence Assay for Intracellular NF-κB Expression

After HepG2 cells (2 × 10^4^ cells/well) were incubated with 1.0 mM OA and 10 μM antioxidant for 24 h in a 96-well culture plate, the cells were washed thrice with ice-cold PBS and then fixed with 4% formaldehyde for 10 min at RT. After washing, the cells were permeabilized by incubation with 100 μL of 0.2% Triton X-100 solution in PBS for 15 min at RT. Thereafter, the cells were incubated with polyclonal antibody for p-NF-κB subunit p65 (Ser 311) (rabbit-polyclonal IgG, Santa Cruz Biotechnology) diluted at a 1% BSA 0.2% Triton X-100 solution and shaking it off for 1 h at RT. Then, the cell was stained with Alexa fluorescent 488 donkey anti-rabbit IgG (H + L) (1:100, Invitrogen) for 1 h. Lastly, the cells were stained with 100 μL DAPI solution (1 μg/mL) (ThermoFisher Scientific) for 5 min and then observed using fluorescent microscopy (Leica DMI6000 B). 

### 4.13. Statistical Analyses 

All the statistical analyses were performed using Microsoft Excel and GraphPad Prism 5 software (GraphPad Software, Inc., La Jolla, CA, USA). Data are presented as mean ± standard deviation (SD) values. One-way analysis of variance (ANOVA) followed by the Bonferroni multiple-comparison test were used to assess the differences between the experimental groups. A *p*-value of < 0.05 was considered statistically significant. An asterisk (*) indicates a significant difference using one-way ANOVA when compared with the negative control. Sharp (#) indicates a significant difference between the experimental groups compared with the positive control (OA-treated and steatosis-induced cells). Dagger (†) indicates a significant difference among the antioxidants using one-way ANOVA.

## Figures and Tables

**Table 1:** Primer sequences for real-time PCR.

Gene	Forward Primer (5′-3′)	Reverse Primer (5′-3′)	Accession Number
*GRP78*	5′-CAACTGGGTCATGTGCATCTG-3′	5′-CTCACCCCACACACGAGTATC-3′	NM_005347.4
*ATF6*	5′-TTACCAGCTACCACCCATAAC-3′	5′-TCTGCTGATCTCGGAGGTAAG-3′	NM_007348.3
*ATF4*	5′-GAGATAGGAAGCCAGACTACACTG-3′	5′-CTCACCCTTTACTTTTGCTGCTACC-3′	NM_001675.4
*XBP1*	5′-CAGCTCAGACTGCCAGAGATC-3′	5′-CAATACCGCCAGAATCCATGG-3′	NM_001079539.1
*GADD34*	5′-CTGGCTGGTGGAAGCAGTAA-3′	5′-TATGGGGGATTGCCAGAGGA-3′	NM_014330.3
*CHOP*	5′-CGAGCTCTGATTGACCGAATG-3′	5′-TGGCACTAGTGAGAGGGTAGT-3′	NM_001195053.1
*Nrf2*	5′-TGCCCTCACCTGCTACTTTAAG-3′	5′-CCACACTGGGACTTGTGTTTAG-3′	NM_001145412.3
*SOD1*	5′-GCGTGGCCTAGCGAGTTA-3′	5′-CCACACCTTCACTGGTCCA-3′	NM_000454.4
*NQO1*	5′-CCTCTATGCCATGAACTTCAATCC-3′	5′-GAACTGGAATATCACAAGGTCTGCG-3′	NM_000903.2
*PPAR* *-γ*	5′-GTGACTCTGCTCAAGTATGGTGT-3′	5′-TGAATCCTTGGCCCTCTGAGATG-3′	NM_001330615.1
*SREBP-1c*	5′-GCAGCCCTGGTCTACCATAA-3′	5′-AATGCAGCCGCCACATAGAT-3′	NM_001005291.2
*ACC-α*	5′-CTGCCTGGGTTTGGGGATAA-3′	5′-GCACCCTCTTCACCCCTTAA-3′	NM_198834.2
*FAS*	5′-AG CTGCTGTGGAAGGATAAC-3′	5′-GCCTTGTCCTGCAGTGTGTAC-3′	NM_004104.4
*SCD-1*	5′-ACGCTTGTGCCCTGGTATTT-3′	5′-TATTCTCCCGGGGGCTAATG-3′	NM_005063.4
*PPAR-α*	5′-CCACCACACTTCCAGAGACC-3′	5′-TCTCATCGGAGCTGGAGGAA-3′	NM_001001928.2
*ACO*	5′-GCCATCACGCTCGGCTAATT-3′	5′-TGAGGTGGCTTGTGGTTA-3′	NM_001185039.1
*CPT-1*	5′-AGTCGGTGAGGCCTCTTATG-3′	5′-CCTCGTCCTCGGAGGTAG-3′	NM_001031847.2
*NF-* *κB*	5′-GATGGAACCACACCCCTGCA-3′	5′-AGTCATCCAGGTCATAGAGAG-3′	NM_001165412.1
*TNF-a*	5′-CATGTTGTAGCAAACCCTCAAG-3′	5′-GAGGACCTGGGAGTAGATGA-3′	NM_000594.3
*IL-1a*	5′-GGCTGCATGGATCAATCTGTG-3′	5′-TCTTCAGAACCTTCCCGTTGG-3′	NM_000575.4
*IL-6*	5′-AACAAATTCGGTACATCCTCGA-3′	5′-ACCAGGCAAGTCTCCTCATTG-3′	NM_000600.4
*IL-8*	5′-GAATTCTCTTGGCTGGCTTCCTTAC-3′	5′-GATGTGCTTTTCGTTGGGGAAGATG-3′	NM_000584.3

## Supplementary Materials

The following are available online at http://www.mdpi.com/1422-0067/19/9/2563/s1.
