# Supplementary material for: Effects of Antioxidants in Reducing Accumulation of Fat in Hepatocyte
Source: Int J Mol Sci. 2018 Aug 29;19(9):2563. doi: 10.3390/ijms19092563 (PMC6164327; doi:10.3390/ijms19092563)
Supplement: Supplementary file 1 [file ijms-19-02563-s001.zip › ijms-334406-SI.pdf]

Int. J. Mol. Sci. 2018, 19, 2563; doi:10.3390/ijms19092563 www.mdpi.com/journal/ijms

Supplementary Data (jims-334406)-3rd revision

(A) (B)

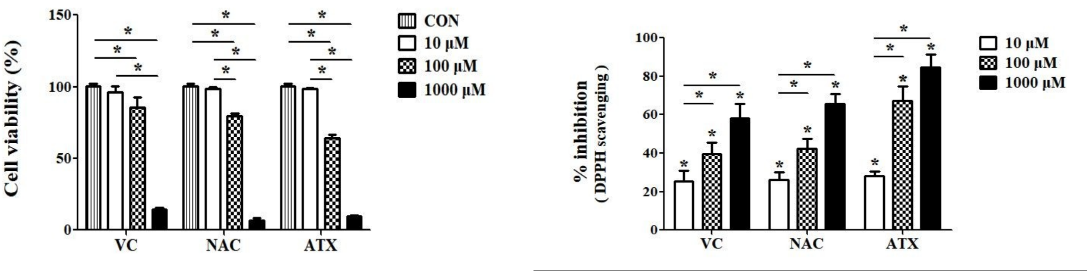

Effects of antioxidant concentrations on cell survival and radicals scavenging ability. Cell viability (%) and DPPH radical scavenging effect (%) of each antioxidant were examined in three kinds of concentration for each antioxidant, 10 μM, 100 μM, and 1000 μM. Cell viability (%) was compared to between not only antioxidant concentration but also three antioxidants in comparison with control (100%) (A). DPPH free radical scavenging effect was also compared to between not only antioxidant concentration but also three antioxidants when it was compared to control DPPH scavenging effect (0% inhibition rate) (B). Data are represented as mean ± SD values (A, $n = 3$; B, $n = 4$). Asterisk (*) indicates a significant difference for antioxidant concentrations and for three kinds of antioxidants ($p < 0.05$).
